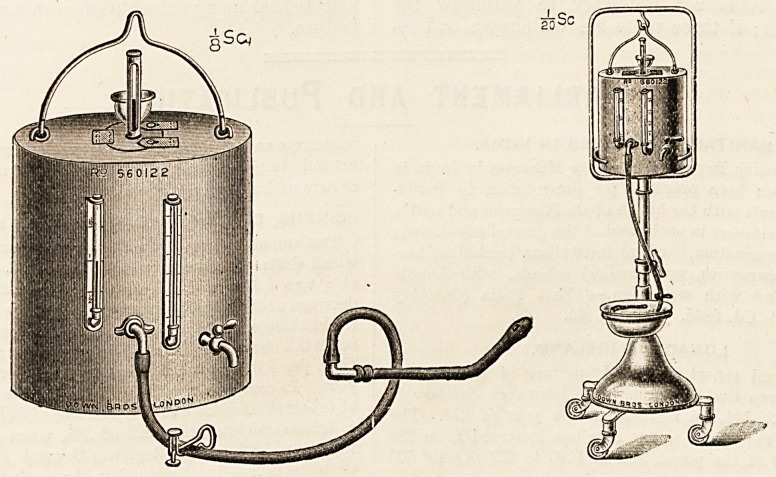# A Note on Continuous Proctoclysis

**Published:** 1910-09-24

**Authors:** E. Wilson Hird

**Affiliations:** House Surgeon to the Gynæcological, Aural, and Ophthalmic Departments, the General Hospital, Birmingham.


### Resident Medical Officers' Department.

### A NOTE ON CONTINUOUS PROCTOCLYSIS.

By E. WILSON HIRD, M.R.C.S., L.R.C.P. Lond., House Surgeon to the Gynæcological, Aural, and Ophthalmic Departments, the General Hospital, Birmingham.

There are two methods of administering continuous rectal saline with which I am acquainted: (1) That form advocated by Dr. Murphy; (2) the "Drop" method.

After reading Dr. J. B. Murphy's classical article[1] on general peritonitis and its treatment, I was particularly impressed with one portion of the treatment he advocated—namely, continuous administration of normal saline solution by the rectum. Ever since the adoption of this form of treatment for general peritonitis at this hospital, it has been my endeavour to obtain a suitable apparatus for its administration. The chief difficulty has been to obtain a vessel which would maintain the temperature of the saline solution at the correct level.

### Murphy's Method.

Before entering into a description of the apparatus I deem it advisable to quote a few points from Dr. Murphy's article, upon which he lays particular emphasis, for success in using Murphy's method depends absolutely upon attention to detail. He says: "The retention of fluid in the colon depends entirely upon the method of its administration. The fluid should be given through a tank to which is attached a ⅜th of an inch bore rubber hose fitted with a hard glass vaginal douche tip with multiple openings. The tube should be flexed almost

[1] "Perforative Peritonitis," by J. B. Murphy, *Journal of Surgery, Gynæcology, and Obstetrics*, June 1908.

to a right angle three inches from the tip. A straight tube must not be used, as the tip produces pressure on the posterior wall of the rectum when the patient is in the Fowler position. The tube is inserted into the rectum to the flexion angle, and secured in place by adhesive strips of plaster to the thigh, so that it cannot come out." Personally I consider glass to be dangerous, especially if the patient is inclined to be restless; a hard rectal tube tends to paralyse the sphincters, and leakage from the bowel results. I have devised a rubber rectal tube which overcomes these objections. Further, he states "that the tank should be suspended from six to fourteen inches above the buttocks, and raised or lowered so as to just overbalance hydrostatically the intra-abdominal pressure, *i.e.* it must be just high enough to require sixty minutes for one pint and a half of saline to flow in—the usual quantity given every two hours.

"The flow must be controlled by gravity alone, and never by a forcep or constriction on the delivery tube, so that when the patient strains to void flatus the fluid can flow rapidly back into the reservoir, otherwise it will be discharged into the bed. It is the ease of flow to and from the bowel that insures against over-distension and expulsion on to the linen. The fountain should be graded, the temperature of the saline maintained at 100° F. The tube should not be removed from the rectum for two or three days. When the nurse complains that the saline is not being retained, it is certain that it is not being properly given."

### The Writer's Experience.

I have tried various devices for giving continuous saline per rectum with a constriction on the delivery tube and an ordinary No. 12 Jaques rubber catheter inserted into the rectum. This constitutes the second method of giving continuous proctoclysis I alluded to. The saline flows into the bowel at the rate of one drop per second; the drop from the eye of the Jaques catheter used is nearly five minims, and this works out at the rate of one pint an hour. Although I have had very good results when adopting this method of administering rectal salines, I have found nevertheless as a general rule that the patient retains the saline well for the first six or twelve hours, but after that time has usually been unable to retain a further quantity; then the saline has to be discontinued for two or three hours and commenced afresh, only to meet with the same difficulty at probably a shorter interval.

### Objections to the Shunt Methods.

In order to overcome this objection I have tried various "shunts," the shunt being introduced at the point where the catheter is joined to the delivery tube. I have found such a device to be quite useless, as the fluid in the bowel does not flow back along the catheter and up the side tube into the empty funnel ready to receive it, but was invariably passed into the bed. Another objection to this method is that it does not matter how good the heat-retaining power of the reservoir is, with a constriction on the delivery tube the rate of flow below the constriction is so slow that the solution in the delivery tube above it is practically stagnant, and soon becomes cold. Even with a delivery tube well insulated with asbestos the result is practically the same, although the saline solution in the reservoir is nearly at boiling point. The saline solution passing into the bowel is well below body temperature. The administration of a large quantity of cold saline solution to a patient suffering from a severe condition like general suppurative peritonitis must be very injurious. With this method I have seen indicators designed, which are fixed in the course of the delivery tube, in order to show that the saline solution is passing into the rectum. I mention this device only to condemn it, as an indicator on the delivery tube is only another means for further heat loss. The drop method of giving saline per rectum is by no means comparable to the method advocated by Dr. Murphy. I remember one patient who was given continuous saline by the drop method, and retained twenty pints a day for three days, but this is the exception rather than the rule. The uses of continuous proctoclysis are by no means confined to the after-treatment of general suppurative peritonitis; I have found this method of giving normal saline solution per rectum very useful in combating shock and collapse after severe abdominal operations. After infusing a patient suffering from post-operative shock or collapse intravenously with normal saline, I have often observed that the volume of the pulse has after a varying interval fallen to the original level, and the blood pressure has also fallen very low. Now in some cases I have found that after infusing intravenously with normal saline and adrenalin until a full pulse was evident at the wrist, if, as soon as the infusion was completed, the patient was immediately given continuous saline per rectum, not only was the pulse volume and blood pressure maintained, but in a few hours' time both were materially increased. In one case of severe post-operative shock and collapse, which I well remember, as it was the first case on which I tried this plan of treatment, the patient was infused intravenously with five pints of normal saline solution with five minims of adrenalin in each pint; as soon as the infusion was completed, she was given continuous saline per rectum. She retained and absorbed twenty-two pints of saline in the first twenty-one hours and made a perfect recovery. Bearing in mind the great tissue waste in prolonged surgical shock, and the fact that the patient has been semi-starved for some hours previous to operation, the store of carbohydrates in the body must be considerably exhausted. I have given a 6 per cent. solution of dextrose continuously by the rectum up to three pints; this represents about 4¾ ounces of the solid sugar, continuing afterwards with normal saline for the first thirty-six or forty-eight hours, stopping the saline after that time if the patient's condition, as judged by the pulse, was satisfactory.

### Some Useful Hints.

It is not necessary to discontinue giving saline per rectum in order to administer nutrient enemata. Patients after operation, especially if suffering from shock or collapse, derive practically no benefit from the exhibition of ordinary nutrient enemata, for little or none of the ingredients of these mixtures are absorbed; practically all the nutrients given are recovered when the patient's rectum is washed out. Unless there are any contra-indications, patients after abdominal section are soon able to take sufficient nourishment by the mouth. In exceptional cases where it is not advisable to give anything by the mouth and the patient needs feeding, one to two ounces of either dextrose or glucose can be added to each pint of saline given, and it is readily absorbed. Continuous saline also relieves thirst, which is so distressing to patients after a surgical operation.

I might mention at this point that an appendicostomy opening offers no advantages over the rectum for the administration of continuous saline, besides possessing many drawbacks.

### Pituitary Extract.

I would like to add a note with regard to the value of pituitary extract in surgical shock; in my experience it offers no advantages over adrenalin, when given either intravenously or injected into the muscles. In some cases the effect on the blood pressure and pulse was not so marked as when adrenalin was employed. In only one case was the blood pressure markedly raised after its exhibition. In this instance the patient suffered from severe griping pains in the abdomen after each injection—not at all a desirable result. In another instance, when the drug was given, the blood pressure steadily declined, although one cubic centimetre of a 20 per cent. solution was given every hour for several doses.

Neither does it to my mind offer any advantages over eserine salicylate when given as a stimulant to peristalsis in general peritonitis. Twelve months ago Mr. Lockhart Mummery mentioned that he himself had not yet been able so far to standardise solutions of the gland satisfactorily for clinical purposes; this may account for the failures I have mentioned.

### The Author's Apparatus.

In conclusion I append a description of the apparatus which I have devised for the administration of continuous rectal salines, with due regard to the essentials enunciated by Dr. Murphy:—

It consists of a metal can of one and a half pints capacity, the interior of which has been prepared so that the saline solution will have no corrosive action upon it. This vessel is surrounded by a hot-water jacket, the water jacket is protected by a thick layer of non-conducting materials. The whole is enclosed in a polished metal case, which is further protected by an outer covering of thick felt. On the front of the apparatus are two glass gauges: one communicates with the interior of the saline can, and is graduated in half-pints, so that the amount of saline entering the rectum can be readily estimated; the other communicates with the interior of the water jacket; by means of it the jacket can be filled accurately without spilling. The capacity of the water jacket is seven and a half pints, and it is filled by means of a funnel fixed on the top of the apparatus; it can be easily emptied by the tap shown on the figure. The aperture of the saline tank is large enough to admit the hand, so that it can be readily cleansed after use. It is closed by a metal lid, which has a rubber core in the centre; through the middle of this core a Fahrenheit thermometer enclosed in a metal case is fixed, so that the temperature of the saline in the reservoir can be easily noted. Both the indicator and the outlet of the saline tank can be removed for cleaning. The saline leaves the can through a delivery tube of three-eighths of an inch bore. This tube is three feet in length, and is connected to a large rubber rectal tube by a glass junction. The apparatus is suspended on an adjustable stand mounted on ball-bearing castors, so that it can be readily wheeled up to the bedside. Messrs. Down Brothers showed me a stand of their own design, which can be raised or lowered by turning a handle; this is an ideal one for the proper working of this apparatus. Once it has been properly adjusted both the saline tank and the hot-water jacket can be replenished without interfering in any way with its proper working. The temperature at which the saline solution and the hot water required to fill the apparatus is kept is 110° F. when used without a constriction on the delivery tube. If a constriction on the delivery tube is used both the saline solution and the water in the jacket must be at 212° F., and the constriction, whether it be screw-clip or forceps, should be placed as near to the outlet as possible. The saline can will need replenishing every hour when the apparatus is used by the drop method, and every two hours when used according to Dr. Murphy's instructions. This is no detriment, for in my experience patients who are given continuous rectal salines are generally so ill that they require attention more often than that. The hot water in the jacket requires to be changed about every two or three hours; in any case it is not necessary to withdraw the whole of it; if three pints are withdrawn and replaced with hot the temperature can by this means be adjusted to the proper level. I have found that the "heat loss" of this apparatus amounts to less than five degrees an hour when used by Murphy's method; if a constriction is used on the tube the loss is much greater, since both the water and the saline have to be used at a very much higher temperature.

I abandoned the idea of using an "electrical heater" to maintain the temperature of the saline solution for two reasons: Electricity, unfortunately, is not installed everywhere, whereas hot water can always be obtained; and the cost of the current required to keep such an apparatus in constant use. Every precaution has been taken to make this apparatus as light and as portable as possible; it has been used by many members of the surgical staff of the General Hospital, Birmingham, and has given every satisfaction.

To Messrs. Down Brothers my thanks are due for the admirable manner in which they have carried out my instructions, to whom at the same time I am indebted for many valuable suggestions in its manufacture.

## Figures and Tables

**Figure f1:**